# American Medical Association

**Published:** 1900-07

**Authors:** 


### EDITORIAL NOTES AND COMMENTS.

The Business office of THE JOURNAL-RECORD is 305, 306 Fitten Building.

The Editorial office is 318-319-320 The Prudential.

Address all Business communications to Dr. M. B. Hutchins, Mgr.

Make remittances payable to THE ATLANTA JOURNAL-RECORD OF MEDICINE.

On matters pertaining to the Editorial and Original Communications address Dr. Bernard Wolff, Atlanta.

Reprints of original articles will be furnished at cost price. Requests for the same should always be made *on the manuscript*.

We will present, post-paid, on request, to each contributor of an original article, twenty (20) marked copies of THE JOURNAL-RECORD containing such article.

### AMERICAN MEDICAL ASSOCIATION.

THE fifty-first annual meeting of the American Medical Association was held at Atlantic City, N. J., June 5–8. Owing to the attractiveness of the place and the season of the year, the meeting was largely attended, there being close to 2,500 members present. The address of the president, W. W. Keen, contained a presentation of the situation of medical matters at present.

The address on Surgery was delivered by Dr. W. L. Rodman of Philadelphia. Other addresses were made by Victor C. Vaughn of Michigan and John A. Witherspoon, of Nashville, Tenn.

The report of the treasurer showed the affairs of the Association to be in a very flourishing condition.

In a short note some time ago in this journal we propounded the query, "Who the deuce is Simmons?" Simmons is the secretary of the Association and the editor of its journal, which, under his wise and progressive administration, has been made to blossom like the rose. Everybody now knows who Simmons is, or ought to know. Hence we rise to withdraw the inquiry.

The name of the Section on State Medicine was changed to Section on Hygiene and Sanitary Science—a very commendable dropping of a term that lived at a time when physiology was called the institutes of medicine and belles-lettres figured professionally in the college curriculum. A new section—that of pathology and bacteriology—was created.

The medal of the Association was awarded to Dr. A. L. Benedict of Buffalo, N. Y., for his paper on "Quantitative Tests for Proteolysis."

The Senn medal was won by Gregory F. Connell with his essay on "Extrophy of the Bladder."

The following resolution, introduced by Dr. Tuckerman of Cleveland, O., was adopted:

"*Resolved*, That the National Legislative Committee, in conjunction with the Special Committee on Reorganization of the Army and Navy Medical Corps, cause to be drafted a bill providing for adequate instruction in hygiene and sanitation in the National Military and Naval Academies, such bill to be perfected at the next meeting of the Joint National Legislative Committee of the American Medical Association and affiliated societies, and to be pushed through Congress as speedily as possible.

The constitutional amendment offered at the last meeting by Dr. Dudley S. Reynolds of Kentucky was also adopted. It is as follows:

"*Provided*, however, that no State, county or other auxiliary body sending representatives shall receive into its membership any one who may after 1901 have received the degree of Doctor of Medicine on less than four years of graded instruction or an equivalent requirement."

The following was also adopted:

"*Resolved*, That the General Executive Committee recommend that the Board of Trustees be requested to approve and pay bills to an amount not exceeding $1,500 annually for the purpose of furnishing an official stenographer for each Section, as well as for the General Executive Committee and the general sessions of the Association, the stenographers to be secured through the Board of Trustees and the Secretary of the Association."

The following officers were elected:

President—Dr. C. A. L. Reed, of Cincinnati, Ohio. Vice-Presidents—Dr. A. W. Calhoun, Atlanta, Ga.; Surgeon A. A. Woodhull, U. S. A.; Dr. Philip Marvel, Atlantic City, N. J.; Dr. Wm. E. Quine, Chicago, Ill. Treasurer—Dr. H. P. Newman. Secretary and Editor—Dr. George H. Simmons. Librarian—Dr. Geo. W. Webster—the last three of Chicago.

Trustees of the *Journal*—Drs. M. F. Porter of Indiana, E. Fletcher Ingalls of Illinois, W. L. Rodman of Pennsylvania, and Jos. W. Matthews of Kentucky.

Judicial Council—Drs. J. R. Guthrie of Iowa, G. B. Gillespie of Tennessee, R. C. Moore of Nebraska, I. J. Heidberger of the District of Columbia, Jno. B. Roberts of Pennsylvania, Chas. S. Rodman of Connecticut, and S. L. Jepson of West Virginia.

The address on Surgery is to be delivered next year by Dr. Jno. A. Wyeth of Philadelphia; on State Medicine, by Dr. Geo. M. Kober of Washington City; and on Medicine, Dr. N. S. Davis, Jr., of Chicago.

The next place of meeting is St. Paul, Minn.

Chairman of Committee of Arrangements—Dr. Jno. F. Fulton of St. Paul.

The election of Dr. A. W. Calhoun is a well-merited honor. Dr. Calhoun has long been eminent in the profession, and has already been distinguished by election to high office in various medical bodies with which he has been connected. None more approve and applaud this last distinction than his fellow practitioners in this city.

An editorial in a late number of the Philadelphia *Medical Journal* suggests that the meeting for 1902 be held in New Orleans in January of that year. We are convinced that any of the large Southern cities will be pleased at the notion of entertaining the Association, and will compete for the privilege in spite of the unkind things that were said of the Atlanta meeting in 1896 by some ungracious critics in certain New England and Western medical journals.

